# Hypothetical versus experienced health state valuation: a qualitative study of adult general public views and preferences

**DOI:** 10.1007/s11136-022-03304-x

**Published:** 2022-11-23

**Authors:** Philip A. Powell, Milad Karimi, Donna Rowen, Nancy Devlin, Ben van Hout, John E. Brazier

**Affiliations:** 1 School of Health and Related Research, University of Sheffield, Regent Court, 30 Regent Street, Sheffield, S1 4DA UK; 2 OPEN Health Evidence & Access, Rotterdam, The Netherlands; 3 Health Economics Unit, School of Population and Global Health, University of Melbourne, Melbourne, Australia

**Keywords:** Experienced health state, General public values, Health-related quality of life, Health state valuation, Hypothetical health state, Qualitative research

## Abstract

**Objectives:**

Responses from hypothetical and experienced valuation tasks of health-related quality of life differ, yet there is limited understanding of why these differences exist, what members of the public think about them, and acceptable resolutions. This study explores public understanding of, opinions on, and potential solutions to differences between hypothetical versus experienced responses, in the context of allocating health resources.

**Methods:**

Six focus groups with 30 members of the UK adult public were conducted, transcribed verbatim, and analysed using framework analysis. Participants self-completed the EQ-5D-5L, before reporting the expected consequences of being in two hypothetical EQ-5D-5L health states for ten years. Second, participants were presented with prior results on the same task from a public (hypothetical) and patient (experienced) sample. Third, a semi-structured discussion explored participants’: (1) understanding, (2) opinions, and (3) potential resolutions.

**Results:**

Twenty themes emerged, clustered by the three discussion points. Most participants found imagining the health states difficult without experience, with those aligned to mental health harder to understand. Participants were surprised that health resource allocation was based on hypothetical responses. They viewed experienced responses as more accurate, but noted potential biases. Participants were in favour of better informing, but not influencing the public. Other solutions included incorporating other perspectives (e.g., carers) or combining/weighting responses.

**Conclusion:**

Members of the UK public appear intuitively not to support using potentially uninformed public values to hypothetical health states in the context of health resource allocation. Acceptable solutions involve recruiting people with greater experience, including other/combinations of views, or better informing respondents.

**Supplementary Information:**

The online version contains supplementary material available at 10.1007/s11136-022-03304-x.

## Introduction

When valuing health-related quality of life (HRQoL), the conventional approach—in the UK and elsewhere—is to have respondents from the general population value *hypothetical* health states [1]. This method stands in contrast to respondents valuing their own *experienced* health state, such as the approach recommended by the Dental and Pharmaceutical Benefits Agency (TLV) in Sweden [2]. The debate on which approach to use—valuing hypothetical or experienced health states—has existed for some time, with arguments levied on either side [3–7]. The choice is of practical significance because hypothetical and experienced values differ, and thus so do conclusions about the perceived severity of health states [8, 9]. Moreover, the size and direction of the difference is not uniform, but varies by health dimension (e.g., mental vs. physical health) [10, 11]. This—hypothetical or experienced—data is used to inform the calculations of quality-adjusted life years (QALYs) utilised in cost-effectiveness analyses, and so ultimately influences health resource allocation (i.e., healthcare funding decisions informed through health technology assessment (HTA)) [12–14].

While there is clear evidence that values derived from hypothetical and experienced health states differ, there is limited research of why these differences exist, what the public think about them, and how they can be resolved [15]. Goodwin et al. (2021) compared the findings of think aloud interviews with patients and members of the public completing health state valuation [15]. This research found differences between the two samples, such as how support from others was appraised and understanding of adaptation. However, this work was not designed to elucidate participants’ understanding, opinions about, or proposed solutions for *differences* observed.

It has been demonstrated that individuals may value health states primarily by assessing the effect of ill health on more fundamental consequences for their lives [16]. Previous qualitative work has highlighted six important consequences: impact on activities, enjoyment, independence, relationships, dignity, and avoiding being a burden [16]. For these consequences, in unpublished data from our research group, it was found that the expectations of the public about life in a health state differs from the experience of individuals in those states. Compared to patients’ experience, the public overestimated the effects of mobility and self-care problems and underestimated the effect of anxiety/depression problems. There is little insight into what members of the public think about such differences, their opinions on asking the public (who may have little or no experience with the health states described) versus people with more experience (e.g., patients), and their views on how differences between the two approaches might be acceptably resolved.

The purpose of this research was to examine and better understand UK public views on the use of hypothetical vs. experienced responses. Using observed data on the experienced and expected (hypothetical) consequences of two health states, we were interested in public views on: (1) the observed differences between experienced and hypothetical/expected consequences for the same health states, (2) the use of experienced and/or hypothetical responses, and (3) perceived acceptable solutions to overcome observed differences. It was hoped this study would reveal the public’s views about whether or not hypothetical preferences should be used to inform policy and, if not, explore solutions that they feel are *most appropriate* for informing policy. Such a contribution to knowledge is important because it helps to inform policy makers in making normative choices about whose preferences should influence decision-making in HTAs and how.

## Methods

Semi-structured focus groups were conducted with the general public, as a recommended method “to clarify, extend, qualify or challenge data collected through other methods” and to facilitate group discussion and consensus [17]. The framework approach was followed as it facilitates a combination of deductive and inductive insights. Framework analysis can be considered a method that combines elements of different research paradigms [18].

### Recruitment and participants

Thirty people across six focus groups (5 people in each) participated. Six focus groups are typically sufficient to identify up to 90% of themes in qualitative analysis [19]. Adult members of the public (aged 18 + years) were recruited by a UK Market Research Agency, stratified to achieve a mix of gender and age. No additional inclusion criteria, such as prior experience with ill health, was imposed. Participants were reimbursed £40.

### Procedure

The focus groups were held at University of Sheffield. The first three groups were run between September and October 2018, with the latter three between February and March 2020 (due to a break in funding). The groups were scheduled for 90 min and facilitated by the lead researcher (PP), and observed by another, both with experience in qualitative and health state valuation methodologies. The researchers had no relationship with participants prior to the focus groups.

**Fig. 1:**
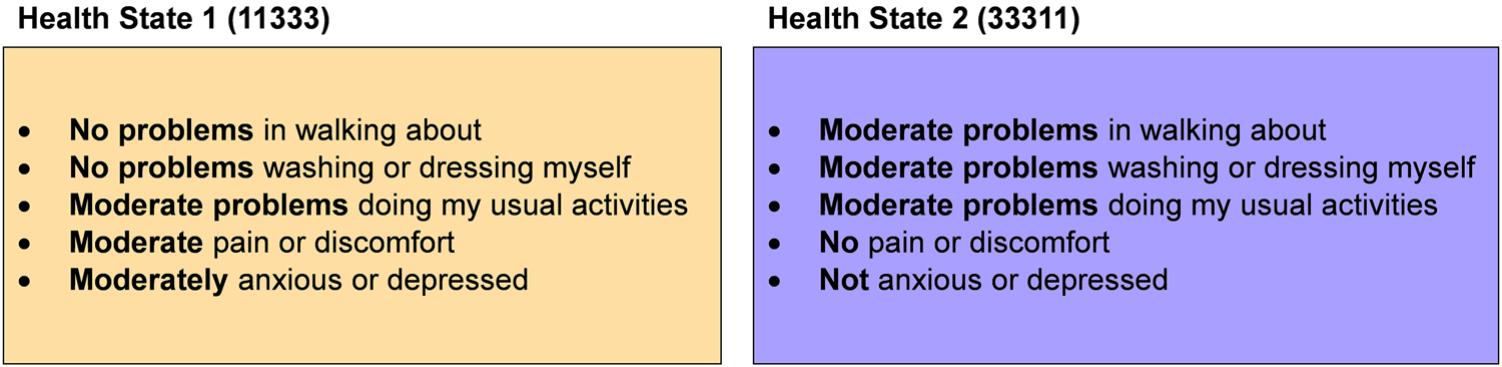
EQ-5D-5L health states used in the study

Following informed consent, each focus group had three stages. First, participants completed a questionnaire (Online Resource 1, Sect. 1) independently, comprising questions on age, gender, and self-reported health on the EQ-5D-5L [20]. This was followed by descriptions of two hypothetical EQ-5D-5L health states (Fig. 1), which participants were asked to imagine living in for 10 years (without change) and report the expected (hypothetical) impact of each state on five consequences, identified as important in prior qualitative work [16]: enjoyment, relationships, independence, dignity, and activities (the remaining sixth consequence ‘avoiding being a burden’ identified in [16] was not included, as this consequence was not available in the data used in the second stage of the focus group). The health states were selected to represent a moderately severe ‘mental health’ state (state 11333) and a moderately severe ‘physical health’ state (state 33311).

Second, participants were given a short presentation (with accompanying handout; Online Resource 1, Sect. 2), describing recently collected unpublished *experienced* data from patients and *hypothetical* data from the public, on the task participants had just completed. In this presentation, differences and similarities in responses were explained, using colour-coded anthropomorphic figures representing the distribution of responses across each sample, rounded to 5% (half figure) or 10% (full figure) (Online Resource 1, Sect. 2). Key messages were: (a) the public tended to overestimate the consequences of the physical health problems relative to patients, (b) the public tended to underestimate the consequences of the mental health problems relative to patients, and (c) the extent of these effects differed by the particular consequence being measured.

Third, an audio-recorded discussion was facilitated using a semi-structured topic guide (Online Resource 1, Sect. 3). This covered three key areas: (1) participants’ own understanding and interpretation of the health states, (2) views on the apparent differences between public hypothetical expectations and patient experiences, and (3) potential acceptable solutions to overcome these differences, in the context of informing healthcare resource allocation. As is common in semi-structured interviews, this topic guide was used to ensure key information was covered, but not all questions were asked in the same way or in the same order.

### Analysis

**Fig. 2:**
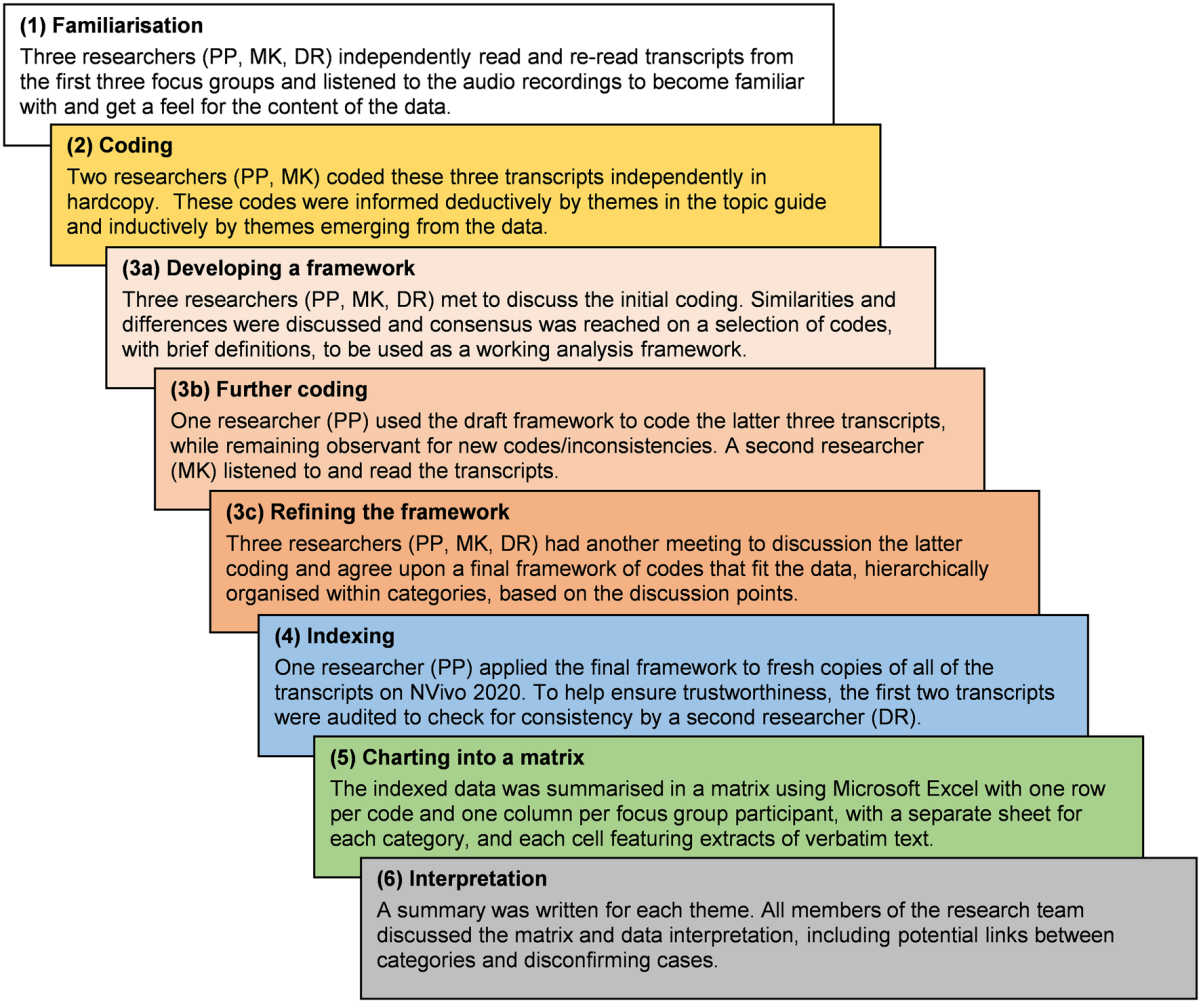
Six-stage framework analysis, adapted from [22]

Questionnaire responses were descriptively summarised. Audio recordings were transcribed verbatim and checked by the researchers. The transcripts were subjected to framework analysis [21], consisting of six stages, described in Fig. 2 [22]. Trustworthiness of the analysis was enhanced in two ways: (i) dual coding and interpretation (multiple researchers were involved in coding and refining the framework), and (ii) auditable decision trail and transparency (all methodological and coding decisions from the raw data to the final framework were recorded) [23].

## Results

**Fig. 3:**
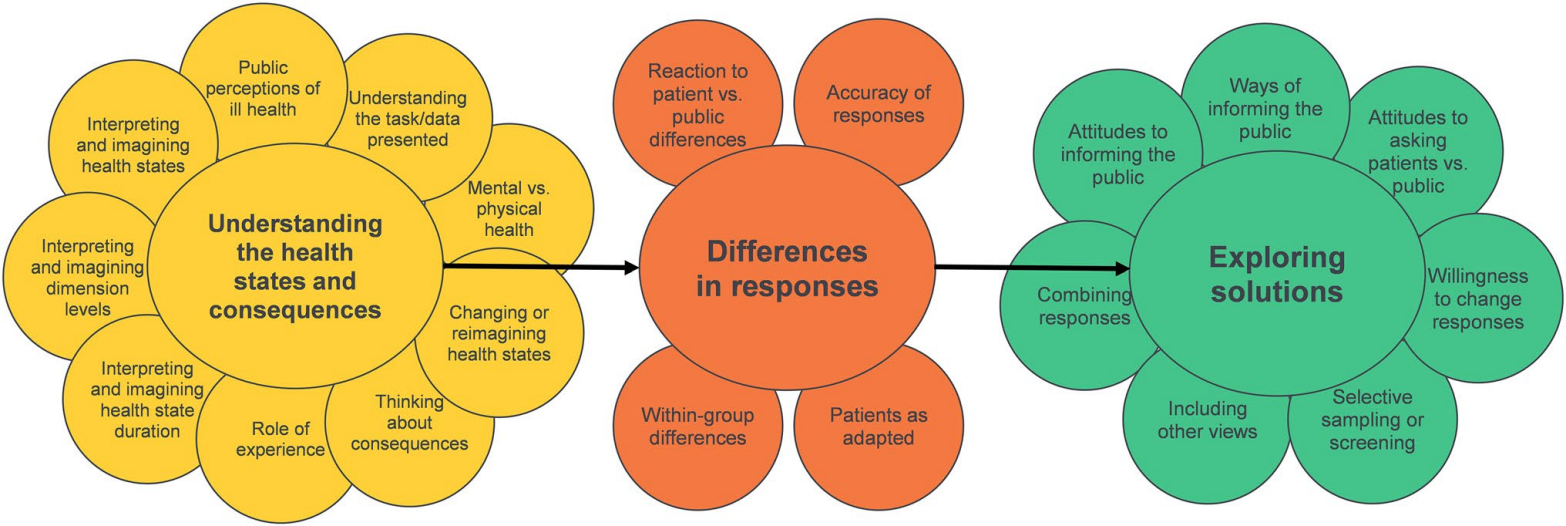
Final qualitative analysis framework

Participants’ background characteristics are in Table 1. Twenty themes emerged from the framework analysis, structured within three categories: understanding the Health States and Consequences (9 themes); Differences in Responses (4 themes); and Exploring Solutions (7 themes). The thematic framework is illustrated in Fig. 3. Table 2 shows coverage in the data. No new themes were added in later focus groups suggesting good data saturation.

**Table 1:** Participant background characteristics

Sex	Male	Female
	14	16
Age	Observed range	Mean (SD)
	18–65	41.17 (12.70)
EQ-5D-5L dimension	Observed range	% reporting any problem
Mobility	1–3	6.66
Self-care	1–3	6.66
Usual activities	1–3	10
Pain/discomfort	1–3	30
Anxiety/depression	1–3	26.66

**Table 2:** Theme coverage and data saturation

Theme	Focus group where theme is indexed						Coverage	
	1	2	3	4	5	6	*N*	Refs
(1) Understanding the health states and consequences								
Changing or reimagining health states	X	X		X	X	X	14	16
Interpreting and imagining dimension levels	X	X	X	X	X	X	20	24
Interpreting and imagining health state duration	X	X	X		X	X	10	11
Interpreting and imagining health states	X	X	X	X	X	X	16	18
Public perceptions of ill health	X	X	X	X	X	X	15	23
Role of experience	X	X	X	X	X	X	23	62
Thinking about consequences	X	X	X	X	X	X	26	33
Understanding mental versus physical health	X	X	X	X	X	X	21	52
Understanding the task and data presented	X	X	X	X	X	X	20	12
(2) Differences in responses between patients and the public								
Accuracy of responses	X	X	X	X	X	X	19	36
Patients as adapted	X	X	X	X	X	X	18	24
Reaction to patient versus public differences	X	X	X	X	X	X	12	12
Within-group differences	X	X	X	X	X	X	20	34
(3) Exploring solutions								
Attitudes to asking patients versus public	X	X	X	X	X	X	23	46
Attitudes to informing the public	X	X	X		X	X	19	29
Combining responses	X	X	X	X	X	X	17	28
Including other views	X	X	X	X	X	X	18	27
Selective sampling or screening	X	X		X		X	6	6
Ways of informing the public	X	X	X	X	X	X	24	60
Willingness to change responses	X	X	X	X	X	X	18	15

Coverage is an estimate of number of people who talked about each theme (‘N’) and how many times the theme was coded (‘Refs’). On occasion a discussion between two or more participants (2 + *N*) could be coded as one instance of a theme in the data (‘Ref’)

### Category 1: understanding the health states and consequences

#### Interpreting and imagining health states

The ability to interpret and imagine living in the health states differed across participants. Participants noted finding the health states difficult to imagine and those that found it easier often reported having had prior experience of aspects of them. Interpretation of individual dimensions within the health states also differed across participants, such as pain and discomfort.

“They’re quite generalised. So if you were to take moderate pain or discomfort, so it could, like you said, it could be anything it could be more of a psychological pain or a physical pain. But there’s nothing to say, for example, if you had back pain or leg pain.” (Participant 1 [P1], Focus Group 2 [FG2]).

#### Interpreting and imagining health state duration

Participants expressed difficulty imagining living in the same health state for 10 years, which was considered a long time. Others noted that their responses would differ if the duration was altered.

“It’s hard to actually imagine yourself being in this situation for ten years. Ten years is a massive amount of time.” (P4, FG2).

#### Interpreting and imagining dimension levels

The severity of problems indicated by the dimension levels (via their corresponding labels) was interpreted differently across participants. Some participants expressed difficulty in understanding the level of problems that a particular label, such as ‘moderate’ would indicate and wanted more detail.

“P4: I have no idea what ‘moderately’ means.

I: What’s ‘moderately’, what’s ‘moderately anxious’ to you?

P2: Very, very, very, very anxious.

I: Is it?

P4: See to me that, moderately anxious is not that anxious.” (FG6).

#### Changing or reimagining health states

Occasionally participants actively re-imagined health states and anticipated change in what was described. Alternatively, participants sometimes reported a perceived incompatibility in the dimension levels, noting, for example, that they would be depressed if they couldn’t walk.

“For me on the second health state I was a bit like if you live with that for 10 years, surely, you’ve got to have a bit of depression, you are a bit anxious, and you will have a bit of pain and, I was a bit like is that entirely true, and like it’s a little bit forced.” (P3, FG4).

#### Role of experience

Experience played a large role in the discussions. Participants generally agreed that you could not really understand a health state unless you had some prior experience of it and to purely imagine it was difficult. They disagreed over the extent of the experience required to facilitate understanding, ranging from observing others in ill health to direct experience. Participants provided examples of their prior experience with similar health states and how that informed their understanding. An interesting discussion was the potential overlap between the 'public' and 'patient' samples, as a number of participants had experience with the health state(s) and thus reported responding like a ‘patient’.

“I think it’s experience and experience of somebody else as well, you know like trying to think of somebody else who has been in maybe similar situations. We’ve probably known maybe older relatives which have, have had to cope with something, you know for. And you think how did they cope or how did they seem? But it’s hard if you’ve not gone through something similar.” (P5, FG3).

#### Public perceptions of Ill health

When discussing their understanding of the health states, participants referred to the public’s understanding of ill health, and particularly mental health, as being distorted in various ways. This included discussing the impact of the media (including social media) on people’s perceptions and a lack of education or exposure.

“They’re just going on, on what they’ve seen on TV really in a way aren’t they? And the media and just getting a perception of it all.” (P3, FG2).

#### Thinking about consequences

Participants described their thought processes regarding consequences of the health state. Sometimes participants interpreted the same consequences differently. For example, with regards to ‘support’, one participant interpreted this as professional support. Participants viewed impact on others/family, financial stability, and dignity, as important consequences of ill health, amongst others.

“I wonder why you’ve not asked about how it would impact on your family and looking after. Like, especially if you’ve got a young family, people have got kids to look after and things like that.” (P4, FG2).

#### Understanding mental versus physical health

Participants’ understanding of the health states differed depending on whether it was more aligned with a mental or physical health problem. Participants thought that mental health problems were more difficult to understand vicariously than physical health problems, as they were more hidden and less talked about.

“I think you can see physical health, mental health can’t see. So, you can easily understand if you’ve got a physical problem, how you’d work, but mental, I’m not sure. So I think we see things differently don’t we?” (P4, FG1).

#### Understanding the task and data presented

Participants’ understanding of the task and data presented (based on a prior study looking at experienced and expected health state consequences), when combined with explanation, was generally favourable. Some participants expressed heterogeneous preferences in how they like to see data presented.

“No, I think that’s fairly self, I got why the red bits were on there before you explained. It was fairly self-explanatory.” (P4, FG6).

### Category 2: differences in responses between patients and the public

#### Accuracy of responses

Overall, participants viewed patients’ responses as more accurate, with the public seen as having got the answers ‘wrong’ in some way. However, participants identified potential biases in both samples, including, for example, that patients may exaggerate or underreport the consequences of their health. Some participants voiced that the accuracy of patient responses may differ by health state, such as people with certain mental health problems (e.g., psychosis) giving distorted responses.

“These are the people that are experienced, that have gone through it or are going through it. So we can pretty much bank this information. Because this is actually, this is factual information. Now compare it with this information and what the public perception cause he’s Mr taxpayer, or they’re Mr taxpayer, but they don't really know, they've seen something or they’ve had a friend or a mate.” (P1, FG4).

#### Reaction to patient versus public differences

Participants were not surprised that there were differences between the patient and public samples, yet some expected an alternative pattern of differences; for example, that the public might think the consequences of mental health problems are worse than physical health problems.

“It doesn’t surprise me, erm, because I think it’s hard to try and visualise being in that mindset, whether it’s a mental health option, or a physical health kind of scenario.” (P1, FG5).

#### Patients as adapted

It was identified that patients may adapt to health states over time and that this may help explain the differences observed. It was suggested that mental health problems may be more difficult to adapt to than physical health problems.

“So obviously that, to me, that shows, it’s quite significant it shows that, you know, people who can’t do things, you can adapt easily and it’s harder to adapt to pain and depression than we think it would be, than people who don’t suffer from it.” (P5, FG3).

#### Within-group differences

Participants argued that people’s responses may be dependent on personal characteristics, independent of experience with the health state. This included age, having children, and people’s personality. This also encompassed perceived generational differences, such as younger people being less resilient.

“It depends on a personality, whether you’re a pessimist or an optimist, I guess. And so if you’ve never had any problems, but if you’re that way about that you’re just so optimistic, you might not think it’s anywhere near as bad, as bad as it is. And vice versa.” (P2, FG1).

### Category 3: exploring solutions

#### Attitudes to asking patients versus public

Participants were generally surprised by the status quo to elicit hypothetical response data from the public. Many were critical of using an *uninformed* public, noting that having some experience of the health state was necessary to provide a realistic response. Some support remained for allowing the public to have a say, but many participants expressed clear support for prioritising asking patients their views to inform policy. The pros and cons of asking patients were discussed, including, for example, patients’ vested interest.

“P1: I think, I think that’s (P1 laughs) like asking members of the public what do you think it’s like to drive that Porsche? We’re not gonna ask the bloke that’s got that Porsche there we are going to ask you what you think it is like to drive that. And they are not, you are going to have an idea ‘oh I think it’s going to be a great acceleration, I think it’s going to handle really well’.

P2: It’s like asking us if we wanna leave the EU (laughter).” (FG4).

#### Combining responses

An interesting approach discussed to inform healthcare funding decisions was the idea of combining responses from patients, the public, and potentially other relevant stakeholders (e.g., carers) into an average, using a weighting system (with various different weightings proposed). This is rather than using one sample or another exclusively.

“I think I’d probably do 50% public, so that you’ve got like your public side, and then 25 patient, 25 carer/relative, and then together that kinda combines the people that are directly affected, or indirectly affected I guess, by it.” (P1, FG5).

#### Including other views

As well as asking patients and the public, participants raised the possibility of including the responses of others. For example, asking family members, healthcare professionals or carers; those considered ‘experts’ in health. This did not necessarily involve combining views.

“The carers (…) surely they’ve got a better perception of how people are living with the conditions that they’ve got. Especially a lot of people with mental illness and stuff.” (P4, FG2).

#### Selective Sampling or Screening

As an alternative to informing the public and/or asking patients, some participants suggested screening or selective sampling, whereby only those that met criteria (e.g., had sufficient experience with the health state) were recruited. Further discussion included the benefits of a broad sampling strategy to ensure representativeness.

“So if you’re gonna ask the public questions I think it should be the public who are directly affected, either been a patient or close to a patient or working within the NHS dealing with those patients. Joe blogs on the street unfortunately, I don’t think.” (P1, FG4).

#### Attitudes to informing the public

Participants were generally in favour of informing the public and thought that it was better than asking an uninformed public. However, reservations were aired about *informing* but not *influencing* people and the potential for the information itself to be biased. Information was not viewed as effective as direct experience in aiding understanding.

“I think that it, it’s, for me, it’s the factual stuff is the inform part (…) like how it does actually effect their day-to-day life and activities (…) and give examples of how it effects, rather than just be sort of someone that sits there and goes it’s awful, my life’s awful, and it really, you know. That’s sort of more influencing and more emotive.” (P2, FG1).

#### Ways of informing the public

Participants shared a wide variety of opinions about ways to inform the public, such as providing additional statistical and factual detail (including examples of what the various dimension levels meant); using media (e.g. video) to enhance engagement; and alternative approaches, such as virtual reality. There was some disagreement over content necessary to help people empathise versus being emotionally influenced to respond a certain way. Some participants suggested using actors or cartoons, and to not use children (because it would be emotionally manipulative). One suggested approach was to provide people with 'a day in the life' of a person living in the health state.

“A day in the life of them isn’t it? Because it’s like what they do, what they get up to, how they manage their day. Because it might make you feel like. If you are seeing someone like, if they are trying to get out of the house and they’re like can’t do it, like you said, your friend were jittery. If you can see it and you think oh shit that’s how they feel, that must be really hard, I think you might feel a little bit differently about it.” (P5, FG4).

#### Willingness to change responses

Despite supporting informing the public, participants differed in the extent they were willing to change their responses based on seeing patients’ responses. This was an interesting contradiction between the participants viewing the patients as more accurate, but being unwilling to change their personal views. Participants were more likely to say they were willing to change their views based on ‘facts’ and were generally resistant to emotional or subjective influences.

“Yeah, no, I would listen to ‘em and if it meant that I changed my mind about something I’d be happy to say, yeah I’ve changed my mind about it. I’m not necessarily saying they would change my mind, but if they did then I would happily admit it.” (P4, FG6).

## Discussion

Despite being debated in the literature for some time [3–7], to our knowledge, this is one of the first studies to qualitatively investigate members of the public’s understanding, views, and preferences on experienced *versus* hypothetical responses in the context of valuing HRQoL and HTA. Several key findings are apparent.

Regarding participant understanding, our sample of the UK public reported finding imagining hypothetical health states and their consequences difficult, without prior experience. This is a common finding [16; 24–26], highlighting known difficulties with purely hypothetical valuation tasks. It was noted that descriptions of mental health problems (i.e., ‘anxious or depressed’) were harder to understand without direct experience than observable physical health problems, which may help to explain the heterogeneous pattern of differences when these different dimensions are valued using experienced versus hypothetical methods [10]. For this sample, experience was central to understanding, and those with experience with the health state(s) reported responding like a ‘patient’. It is thus important to acknowledge that a public sample will include people with experience of the health condition (or ‘patients’) [27], but not purposively recruited on this criterion.

Concerning observed differences, participants viewed patients’ responses as most accurate, but noted potential biases from both samples. There was minimal surprise from participants at the differences observed and as well as viewing the public’s responses as inaccurate, they raised the issue of adaptation in those with experience [9; 28; 29]. It has been outlined that adaptation can be used as an argument for and against using experienced responses, depending on whether adapted or preadapted responses are viewed as more accurate [5]. In this sample, it tended to be the former.

Regarding solutions, most participants were surprised with the status quo in the UK of obtaining values to inform health resource allocation from hypothetical responses. They were unaware that this process occurred. Many were critical of the idea of using a potentially uninformed public and felt that at least some experience with the health state was essential in providing a realistic response. There was general agreement that information about patient experience could be used to *inform* the views of the general public, but not *influence* them, with some discussion over what this distinction meant in terms of informative vs. influencing content, with ‘factual’ and emotive content typically distinguishing the former from the latter. Participants differed in their preferred medium and content for informing the public, with suggestions ranging from factual information to meeting patients face-to-face. However, virtually all participants agreed that more information was needed for them to provide accurate and useful responses to tasks where they are asked to put themselves in hypothetical states. Further ideas involved incorporating other perspectives (e.g., carers), selective sampling, and/or combining and weighting responses from different groups.

There is a growing literature on the use of experienced versus hypothetical valuations [3; 5; 30]. The review by Helgesson et al. (2020) suggests that support for the two positions is distinguished by several unique arguments. The case for using patients’ experienced valuations is made by appealing to theoretical reasons based on welfare economics, while hypothetical valuations from the general public are justified by arguments about the importance of social values (e.g., incorporating the preferences of taxpayers funding the healthcare system), reduced bias, and practical advantages (i.e., easier and less resource intensive sampling). A key implication of this study is that although researchers frame the strength of hypothetical valuations as being justified by social values, the public do not support patient values being ignored. Instead, a principal finding—which appears not to have been previously examined or reported in the literature—is that members of the public appear intuitively not to support the current widespread practice of using values derived from a potentially uninformed public’s responses to hypothetical health states to inform health resource allocation.

Two avenues for further research can be suggested. Research could further examine the solutions proposed around informing members of the general population in valuation exercises (e.g., using information that is ‘factual’ and minimises emotion) [31]. Research could also examine the novel solution of using both public and patient values to generate joint or combined value sets to score preference-based measures [6]. To do so it could first be investigated what aspect(s) of patient and what aspect(s) of general population values are deemed to be the most ‘accurate’.

Study limitations include that the sample was from a single country, the UK, and one geographical location, potentially limiting transferability. In addition, the focus groups were conducted in two time periods, 16 months apart (due to a break in funding), and views may have changed over time. Further, initial themes identified may have influenced and restrictively shaped later focus group discussions. However, care was taken to talk about less explored ideas in later groups and saturation was reached in our analyses, suggesting that all themes were captured for this sample over the research period. Another limitation is that one consequence raised in the literature (dignity) was not included in this study, since we did not have data available on this consequence. Finally, while care was taken to present both sides of the argument to participants, we cannot discount that the way information was presented may have influenced responses, including what was *not* said.

## Conclusion

In conclusion, our findings suggest that the status quo—in the UK and elsewhere—of basing HRQoL values on general public responses to hypothetical health states, to help inform health resource allocation, may not be supported by the public themselves. However, problems were also recognised with using solely experience-based preferences. Instead, properly informing but not influencing the public prior to valuation exercises, selective sampling for experience, and/or combining public hypothetical and experienced responses were viewed as acceptable resolutions by the public.

## Supplementary Information

Below is the link to the electronic supplementary material.

Supplementary file1 (PDF 1756 KB) Online Resource 1. Supplementary file containing: Section 1) Questionnaire given to participants during stage 1 of the focus groups; Section 2) Handout used for stage 2 of the focus groups; and Section 3) Focus group topic guide (.pdf)

## Data Availability

Supporting data is available upon reasonable request, taking into account privacy and ethical concerns.
