# Supplementary material for: Hypothetical versus experienced health state valuation: a qualitative study of adult general public views and preferences
Source: Qual Life Res. 2022 Nov 23;32(4):1187–97. doi: 10.1007/s11136-022-03304-x (PMC10063498; doi:10.1007/s11136-022-03304-x)
Supplement: Supplementary file 1 — Supplementary file1 (PDF 1756 KB) Online Resource 1. Supplementary file containing: Section 1) Questionnaire given to participants during stage 1 of the focus groups; Section 2) Handout used for stage 2 of the focus groups; and Section 3) Focus group topic guide (.pdf) [file 11136_2022_3304_MOESM1_ESM.pdf]

Article Title: Hypothetical versus experienced health state valuation: A qualitative study of adult general public views and preferences

Journal Name: Quality of Life Research

Authors: Philip A. Powell, Milad Karimi, Donna Rowen, Nancy Devlin, Ben van Hout, John E. Brazier

Corresponding Author: Philip A. Powell, University of Sheffield, UK, p.a.powell@sheffield.ac.uk

Online Resource 1

Section 1 - Questionnaire given to participants during stage 1 of the focus groups

University of Sheffield

School of Health and Related Research

Assessing health expectations

This questionnaire contains questions which ask about aspects of your health and about you.

You will then be presented with 2 imaginary descriptions of health scenarios. For each health scenario we will ask you 7 questions about what you imagine life is like in that health scenario.

Please read each question and consider your answers carefully. For each question, please read <u>all</u> answers and select <u>one</u> answer. There are no right or wrong answers; what we want is your opinion.

Before making your choices, please consider the questions carefully. It is important to us that you answer these questions as truthfully and honestly as possible during the whole study. This will allow researchers to make the most use of your provided response to improve methods used to improve health services.

Thank you for completing this survey.

<u>Please answer the following information about yourself.</u>

1. **Are you:**

Male [ ]

Female [ ]

2. **What is your age (in years)?** ☐

By placing a tick in one box in each group below, please indicate which statements best describe your own health TODAY.

3. Mobility

I have no problems in walking about ☐

I have slight problems in walking about ☐

I have moderate problems in walking about ☐

I have severe problems in walking about ☐

I am unable to walk about ☐

4. Self-Care

I have no problems washing or dressing myself ☐

I have slight problems washing or dressing myself ☐

I have moderate problems washing or dressing myself ☐

I have severe problems washing or dressing myself ☐

I am unable to wash or dress myself ☐

5. Usual Activities (e.g. work, study, housework, family or leisure activities)

I have no problems doing my usual activities ☐

I have slight problems doing my usual activities ☐

I have moderate problems doing my usual activities ☐

I have severe problems doing my usual activities ☐

I am unable to do my usual activities ☐

6. Pain / Discomfort

I have no pain or discomfort ☐

I have slight pain or discomfort ☐

I have moderate pain or discomfort ☐

I have severe pain or discomfort ☐

I have extreme pain or discomfort ☐

7. Anxiety / Depression

I am not anxious or depressed ☐

I am slightly anxious or depressed ☐

I am moderately anxious or depressed ☐

I am severely anxious or depressed ☐

I am extremely anxious or depressed ☐

Living life in ill health

In the following questions you will be presented with imaginary descriptions of health scenarios.

In previous studies, people have mentioned that ill health affects them in a variety of ways. In particular, it affects their lives in the following ways: enjoyment, relationships, independence, dignity, activities, and whether they are a burden on other people. We want to better understand how you think ill health would affect your life.

We will ask you to tell us how you think different health scenarios affect these life domains. For each health scenario we will ask you six questions about what you imagine life is like in that health scenario.

Health state 1

Imagine you, yourself, were in the health scenario described below and this health scenario was starting today. When answering the questions please imagine that you will experience each health scenario for the period shown without change. Please imagine that you will have no other health problems besides what is indicated. Please also imagine that death will be very swift and completely painless. Please imagine that you would be in this health scenario even if you were receiving treatment or medication.

You live for 10 years with the following:
• **No problems** in walking about<br>• **No problems** washing or dressing myself<br>• **Moderate problems** doing my usual activities<br>• **Moderate** pain or discomfort<br>• **Moderately** anxious or depressed

Now, imagine you are living in that health scenario and imagine what your life would be like. Imagine a typical day in that life. For that day please answer the following question. If you lived in the health state above:

8. On a typical day, would you cut down the amount of time you spent on work or other activities as a result of your PHYSICAL health?

None of the time	☐
A little of the time	☐
Some of the time	☐
Most of the time	☐

All of the time ☐

9. On a typical day, would you cut down the amount of time you spent on work or other activities as a result of your EMOTIONAL problems (such as feeling depressed or anxious)?

None of the time ☐

A little of the time ☐

Some of the time ☐

Most of the time ☐

All of the time ☐

10. On a typical day, please indicate which statements would best describe your overall love, friendship, and support?

I can have a lot of love, friendship, and support ☐

I can have quite a lot of love, friendship, and support ☐

I can only have a little love, friendship, and support ☐

I cannot have any love, friendship, and support ☐

Remember, you live for 10 years with the following:

- **No problems** in walking about
- **No problems** washing or dressing myself
- **Moderate problems** doing my usual activities
- **Moderate** pain or discomfort
- **Moderately** anxious or depressed

11. On a typical day, please indicate which statements would best describe your overall independence?

I am able to be completely independent	☐
I am able to be independent in many things	☐
I am only able to be independent in a few things	☐
I am unable to be at all independent	☐

12. On a typical day, how much of a burden would you feel you are to other people?

Not at all	☐
A little	☐
A moderate amount	☐
A lot	☐
Totally	☐

13. On a typical day, overall how happy would you feel?

Not at all happy										Completely happy
0	1	2	3	4	5	6	7	8	9	10

Health state 2

Imagine you, yourself, were in the health scenario described below and this health scenario was starting today. When answering the questions please imagine that you will experience each health scenario for the period shown without change. Please imagine that you will have no other health problems besides what is indicated. Please also imagine that death will be very swift and completely painless. Please imagine that you would be in this health scenario even if you were receiving treatment or medication.

You live for 10 years with the following:
• **Moderate problems** in walking about<br>• **Moderate problems** washing or dressing myself<br>• **Moderate problems** doing my usual activities<br>• **No** pain or discomfort<br>• **Not** anxious or depressed

Now, imagine you are living in that health scenario and imagine what your life would be like. Imagine a typical day in that life. For that day please answer the following question. If you lived in the health state above:

14. On a typical day, would you cut down the amount of time you spent on work or other activities as a result of your PHYSICAL health?

1: None of the time	☐
2: A little of the time	☐
3: Some of the time	☐
4: Most of the time	☐

5: All of the time ☐

15. On a typical day, would you cut down the amount of time you spent on work or other activities as a result of your EMOTIONAL problems (such as feeling depressed or anxious)?

1: None of the time ☐

2: A little of the time ☐

3: Some of the time ☐

4: Most of the time ☐

5: All of the time ☐

16. On a typical day, please indicate which statements would best describe your overall love, friendship, and support?

I can have a lot of love, friendship, and support ☐

I can have quite a lot of love, friendship, and support ☐

I can only have a little love, friendship, and support ☐

I cannot have any love, friendship, and support ☐

Remember, you live for 10 years with the following:

- **Moderate problems** in walking about
- **Moderate problems** washing or dressing myself
- **Moderate problems** doing my usual activities
- **No** pain or discomfort
- **Not** anxious or depressed

17. On a typical day, please indicate which statements would best describe your overall independence?

I am able to be completely independent	☐
I am able to be independent in many things	☐
I am only able to be independent in a few things	☐
I am unable to be at all independent	☐

18. On a typical day, how much of a burden would you feel you are to other people?

Not at all	☐
A little	☐
A moderate amount	☐
A lot	☐
Totally	☐

19. On a typical day, overall how happy would you feel?

Not at all happy										Completely happy
0	1	2	3	4	5	6	7	8	9	10

Section 2 - Handout used for stage 2 of the focus groups

Patient and public comparison - instructions

You have already viewed two health states and told us how you think life in those states would be. We will now show you the results of a study in the UK where we interviewed members of the public, like you, and compared their answers to those of people with health problems (who we call ‘patients’). On the following pages, you will see, for both health states, the average expectations of the public and the average experiences of the people in ill health.

Here are the health states again:

- **No problems** in walking about
- **No problems** washing or dressing myself
- **Moderate problems** doing my usual activities
- **Moderate** pain or discomfort
- **Moderately** anxious or depressed

- **Moderate problems** in walking about
- **Moderate problems** washing or dressing myself
- **Moderate problems** doing my usual activities
- **No** pain or discomfort
- **Not** anxious or depressed

On each page you will see a comparison in a table. For each health state, the table will show the average responses if we had asked 10 members of the public their **expectations** about the health state and 10 patients to report what they **experienced** in the health state.

Comparison 1

On a typical day, would you cut down the amount of time you spent on work or other activities as a result of your PHYSICAL health?				
	• **No problems** in walking about<br>• **No problems** washing or dressing myself<br>• **Moderate problems** doing my usual activities<br>• **Moderate** pain or discomfort<br>• **Moderately** anxious or depressed		• **Moderate problems** in walking about<br>• **Moderate problems** washing or dressing myself<br>• **Moderate problems** doing my usual activities<br>• **No** pain or discomfort<br>• **Not** anxious or depressed	
	**Public**	**Patient**	**Public**	**Patient**
None of the time	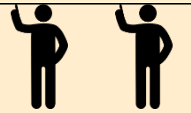	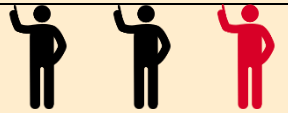	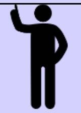	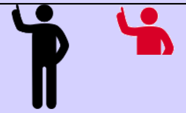
A little of the time	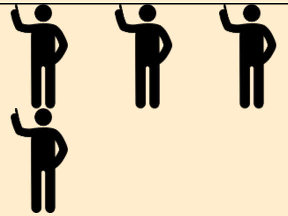	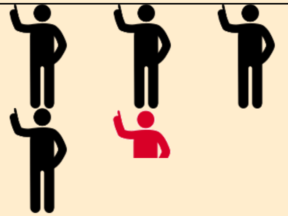	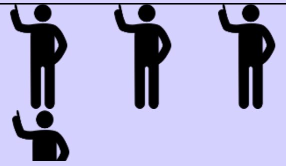	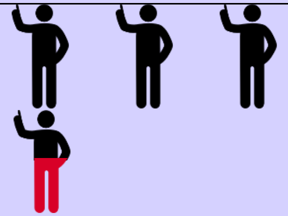
Some of the time	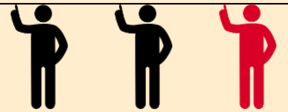	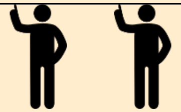	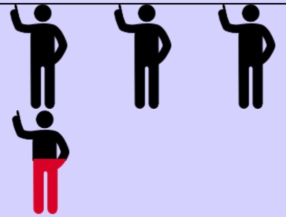	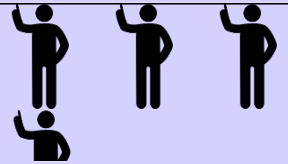
Most of the time	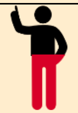	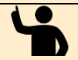	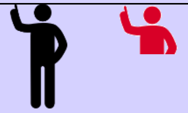	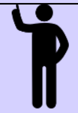
All of the time				

On a typical day, would you cut down the amount of time you spent on work or other activities as a result of your PHYSICAL health?		
	• **No problems** in walking about<br>• **No problems** washing or dressing myself<br>• **Moderate problems** doing my usual activities<br>• **Moderate** pain or discomfort<br>• **Moderately** anxious or depressed	
	**Public**	**Patient**
None of the time	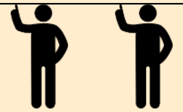	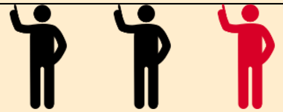
A little of the time	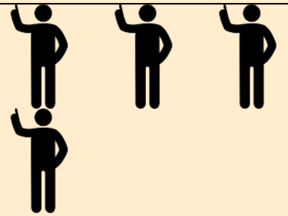	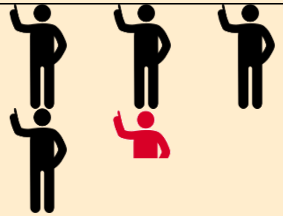
Some of the time	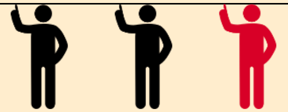	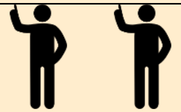
Most of the time	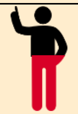	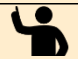
All of the time		

For this health state, on average:

- 2 members of the public expected to cut down *none of the time* spent on work or other activities, but 3 patients reported experiencing this.
- 4 members of the public expected to cut down *a little of the time* spent on work or other activities, but 4.5 patients reported experiencing this.
- 3 members of the public expected to cut down *some of the time* spent on work or other activities, but 2 patients reported experiencing this.
- 1 member of the public expected to cut down *most of the time* spent on work or other activities, but 0.5 patients reported experiencing this.

When you see half a figure, this represents half a person. When you see a red person, this represents a difference between what the public expected and what the patients reported, based on the extra members of the public or patients who gave this response. You see that for this health state patients reported less cutting down in time for work or other activities than the public expected.

Comparison 2

On a typical day, would you cut down the amount of time you spent on work or other activities as a result of your EMOTIONAL problems (such as feeling depressed or anxious)?				
	• **No problems** in walking about<br>• **No problems** washing or dressing myself<br>• **Moderate problems** doing my usual activities<br>• **Moderate** pain or discomfort<br>• **Moderately** anxious or depressed		• **Moderate problems** in walking about<br>• **Moderate problems** washing or dressing myself<br>• **Moderate problems** doing my usual activities<br>• **No** pain or discomfort<br>• **Not** anxious or depressed	
	**Public**	**Patient**	**Public**	**Patient**
None of the time	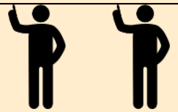	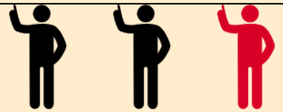	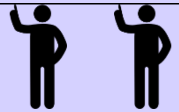	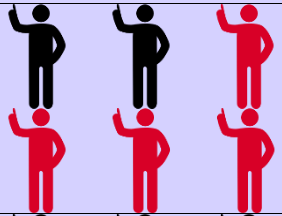
A little of the time	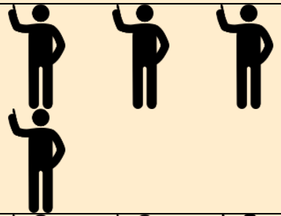	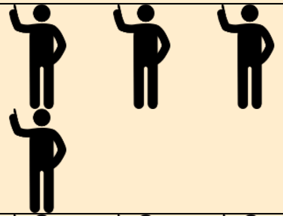	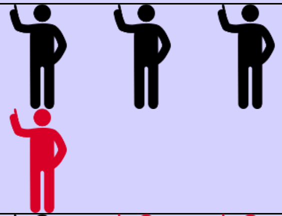	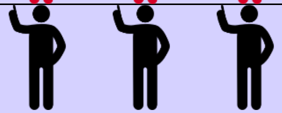
Some of the time	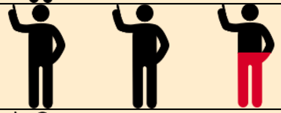	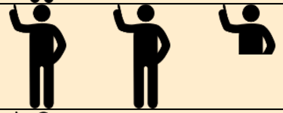	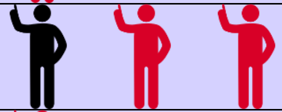	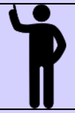
Most of the time	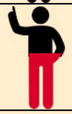	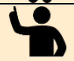	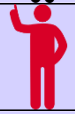	
All of the time				

Comparison 3

On a typical day, please indicate which statements would best describe your overall love, friendship, and support?				
	• **No problems** in walking about<br>• **No problems** washing or dressing myself<br>• **Moderate problems** doing my usual activities<br>• **Moderate** pain or discomfort<br>• **Moderately** anxious or depressed		• **Moderate problems** in walking about<br>• **Moderate problems** washing or dressing myself<br>• **Moderate problems** doing my usual activities<br>• **No** pain or discomfort<br>• **Not** anxious or depressed	
	**Public**	**Patient**	**Public**	**Patient**
a lot of love, friendship and support				
quite a lot of love, friendship and support				
a little love, friendship and support				
cannot have any love, friendship and support				

Comparison 4

On a typical day, please indicate which statements would best describe your overall independence?				
	• **No problems** in walking about<br>• **No problems** washing or dressing myself<br>• **Moderate problems** doing my usual activities<br>• **Moderate** pain or discomfort<br>• **Moderately** anxious or depressed		• **Moderate problems** in walking about<br>• **Moderate problems** washing or dressing myself<br>• **Moderate problems** doing my usual activities<br>• **No** pain or discomfort<br>• **Not** anxious or depressed	
	**Public**	**Patient**	**Public**	**Patient**
completely independent				
independent in many things				
independent in a few things				
unable to be at all independent				

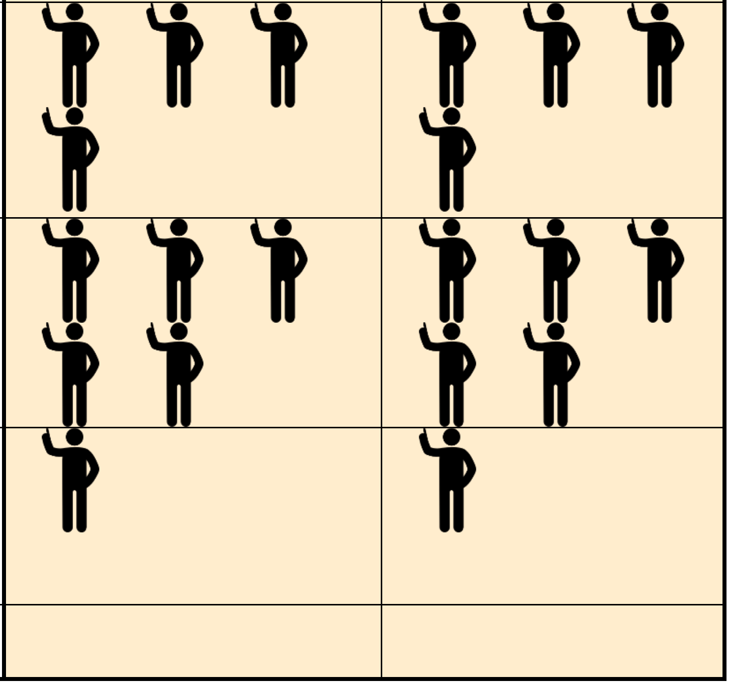

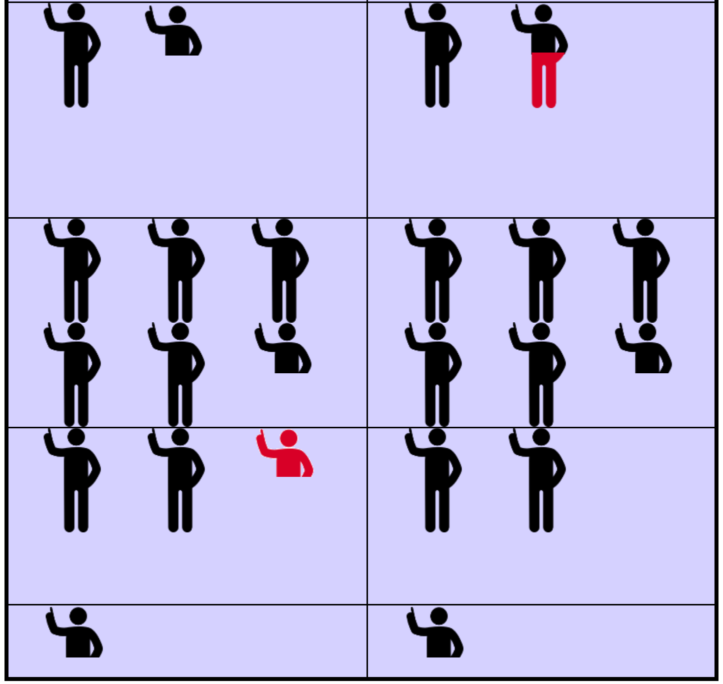

Comparison 5

On a typical day, how much of a burden would you feel you are to other people?				
	• **No problems** in walking about<br>• **No problems** washing or dressing myself<br>• **Moderate problems** doing my usual activities<br>• **Moderate** pain or discomfort<br>• **Moderately** anxious or depressed		• **Moderate problems** in walking about<br>• **Moderate problems** washing or dressing myself<br>• **Moderate problems** doing my usual activities<br>• **No** pain or discomfort<br>• **Not** anxious or depressed	
	**Public**	**Patient**	**Public**	**Patient**
Not at all				
A little				
A moderate amount				
A lot				
Totally				

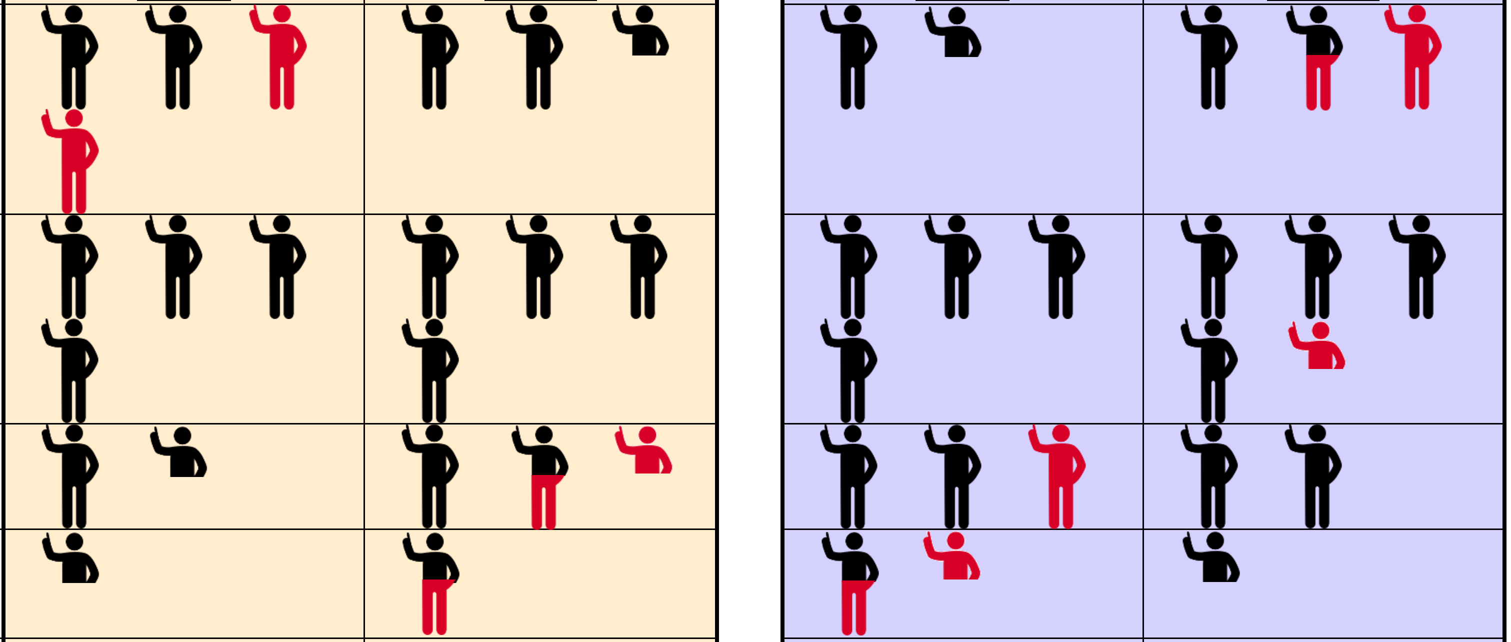

Comparison 6

On a typical day, overall how happy would you feel?				
	• **No problems** in walking about<br>• **No problems** washing or dressing myself<br>• **Moderate problems** doing my usual activities<br>• **Moderate** pain or discomfort<br>• **Moderately** anxious or depressed		• **Moderate problems** in walking about<br>• **Moderate problems** washing or dressing myself<br>• **Moderate problems** doing my usual activities<br>• **No** pain or discomfort<br>• **Not** anxious or depressed	
	**Public**	**Patient**	**Public**	**Patient**
9 to 10 out of 10<br><br>*10 = completely happy*				
8 out of 10				
6 to 7 out of 10				
0 to 5 out of 10<br><br>*0 = not at all happy*				

Section 3 – Focus group topic guide

<table>
<tr><th><u>Stage of focus group</u></th><th><u>Interviewer guidelines</u></th></tr>
<tr><td>Introduction</td><td>Provide and discuss information sheet and consent form

Complete consent form

Ask permission to audio record

Introduce researchers</td></tr>
<tr><td>Explain background of research</td><td>There is a lot of interest in understanding how important different aspects of health are. Understanding what people think is important in terms of their health may be used to make decisions on how to allocate money in the NHS and for some public services. The overall aim of this project is to find out what people think about when considering how important different aspects of health are.

We will ask you a series of questions that involve you imagining living in certain health states. Moderator will briefly explain:

1) Explain briefly that we allocate health resources based on how badly people report that living in a particular health state is. This can be done in a number of ways, by asking members of the general public, which is what is currently done, or patients living in particular health states.
2) Explain the aims of the research – to understand how people value health states, for example on the basis of their consequences in different areas of their life, and how, if at all, this differs between patients and the public
3) Explain that we ask them to fill in a brief questionnaire - to give them an idea of how we collect data on how people view different health states
4) Explain the structure of the group discussion – to look at the results from a larger study into this topic, and to move into a group discussion into suggested reasons for the findings, what the implications are, and where there may be differences between patients and public preferences, and what their views are on approaches to reconcile those differences
5) Explain that the overall goal is to help decision makers make decisions.

Please complete this booklet now.</td></tr>
<tr><td>[Replication of Quantitative questionnaire part 1 to 6]</td><td>See “Survey design for qualitative study.docx”</td></tr>
<tr><td></td><td>**TURN ON RECORDER**</td></tr>
</table>

Showing participants the experienced data	You have spent some time considering what you expect life in some health states to be like. Recently people in a range of health conditions have told us how these health states that you have just been imagining have actually affected their life.<br><br>Here you will see what a patient in that health state experienced and what average members of the public expected. There are some differences between what the average member of the public thought it would be like and what the patient experienced. This does not mean that anyone is wrong. We want to explore these differences together.<br><br>[Handout – "Patient and public results"]
Why we do this?	One reason we explore these differences is because we can ask patients or the public about important aspects of health. If their answers differ then this may affect what services are funded. That is why we are investigating potential differences between the public and patients.<br><br>Moderator is to emphasise that we look for views of the public on what should be done
Stage 1: Understanding of health states	**1. Do you think you understood what it would be like to live in the health states?**<br>• Was the health state easy or difficult to understand? Why was that?<br>• Were you able to imagine yourself in that health state? Was it difficult or easy to do that? Why?<br>• How did you interpret the different aspects of the health state, did you focus on certain words?<br>**2. What did you think about when you were trying to imagine living in the health states?**<br>• How did you go about imagining living with ill health?<br>• What kind of things were you imagining when you read the consequences of living in that health state?<br>• What were your main considerations when giving your answers? For example, were there any important life events or circumstances driving your responses? Did any of you draw on personal experiences or experiences of your family/friends? Did you have previous experience of receiving or seeking support from your environment when being ill?<br>• Were there any other consequences on your life that you thought about when trying to imagine living in that health state, aside from the six questions we asked?
Stage 2: Understanding of differences between patients and the public	**3. As you have seen there are differences between how people experiencing the health states answered the questions and how members of the general public answered the questions. What do you think about the differences?**<br>• Are you surprised by any of the differences?<br>• What do you think explains any of the differences?

	• How far removed do you feel from a patient in that particular health state? Do you think that affected your response?<br>• Which set of views do you think is more accurate? And why?<br>**4. Do you think the differences are problematic?**<br>• Should your views be informed by patient experiences?<br>**5. Do you think differently about the health states, now that you have seen that people experiencing the health state viewed it differently to people like you?**<br>• Would you want a chance to change your answers?<br>• Has it changed your views?<br>• What effect does it have to see the patient values? How would your answers change if you had seen the patient values?
Stage 3: Understanding solutions	**6. In the UK we make decisions about how money in healthcare is spent using what the public think about health states rather than what people experiencing the health state tell us it is like. What do you think we should do, given the differences you have just seen?**<br>• E.g. should we continue to use the public's views?<br>• E.g. should we be trying to make the public more informed?<br>• E.g. should we be applying a weighting to consider patients and the public views? What weight would you give to each and why?<br>• What could a consultation of patients look like?<br>• What should be the role of patients in informing the public's views?<br>• Do you think people would change their views with input from patients?<br>• Which approaches do you find most acceptable? Why is this? Is there anything you would not recommend?<br>**7. How could you make patient views understandable to the public, to people who don't know what it is like to live in the health state?**<br>• Was the presentation of the results understandable today?<br>• Are other formats more appropriate? (videos, drama group, personal reflections, as more formal statistics, graphs, etc.)<br>• Did you find any aspects of the exercise unrealistic? Which bits?<br>• What did you find most difficult in this exercise?<br>• Was there anything missing from the survey on health consequences?
Conclude	Thank and ask for completed quantitative questionnaires and debrief
